# First report of de novo assembly and annotation from brain and blood transcriptome of an anadromous shad, *Alosa sapidissima*

**DOI:** 10.1186/s12863-022-01043-z

**Published:** 2022-03-28

**Authors:** Kishor Kumar Sarker, Liang Lu, Junman Huang, Tao Zhou, Li Wang, Yun Hu, Lei Jiang, Habibon Naher, Mohammad Abdul Baki, Anirban Sarker, Chenhong Li

**Affiliations:** 1 Shanghai Universities Key Laboratory of Marine Animal Taxonomy and Evolution, Shanghai Ocean University, Shanghai, 201306 China; 2 Shanghai Collaborative Innovation for Aquatic Animal Genetics and Breeding, Shanghai Ocean University, Shanghai, 201306 China; 3 Department of Zoology, Jagannath University, Dhaka, 1100 Bangladesh

**Keywords:** *Alosa sapidissima*, De novo transcriptome, Brain & Blood, Annotation

## Abstract

**Objectives:**

American shad (*Alosa sapidissima*) is an important migratory fish under Alosinae and has long been valued for its economic, nutritional and cultural attributes. Overfishing and barriers across the passage made it vulnerable to sustain. To protect this valuable species, aquaculture action plans have been taken though there are no published genetic resources prevailing yet. Here, we reported the first de novo assembled and annotated transcriptome of *A. sapidissima* using blood and brain tissues.

**Data description:**

We generated 160,481 and 129,040 non-redundant transcripts from brain and blood tissues. The entire work strategy involved RNA extraction, library preparation, sequencing, de novo assembly, filtering, annotation and validation. Both coding and non-coding transcripts were annotated against Swissprot and Pfam datasets. Nearly, 83% coding transcripts were functionally assigned. Protein clustering with clupeiform and non-clupeiform taxa revealed ~ 82% coding transcripts retained the orthologue relationship which improved confidence over annotation procedure. This study will serve as a useful resource in future for the research community to elucidate molecular mechanisms for several key traits like migration which is fascinating in clupeiform shads.

## Objective

*Alosa sapidissima* is well discussed among the alosines for its biological, nutritional, and commercial calibre [1–4]. Their native range from the North Atlantic coast extends to several freshwater tributaries where come to reproduce by migrating, sometimes up to 1800 km upstream [5–7]. For high fecundity, marketable weight, and sport fishing, this anadromous fish receives an overwhelming demand, which drives up the exploitation. Numerous obstructions on their passage are limiting free movement and segregating the populations into patches [8–12]. Being sensitive to environmental changes, several reports have anticipated the extinction of shad species namely *Tenualosa. reevesii*, *T. thibaudeaui*, and *Alosa killarnensis* [13, 14]. Considering this risk, American shad restoration project and captive rearing has been undertaken in the USA and China, respectively. Despite these efforts, there is no large scale molecular information published to explain key traits that can strengthen a recovery program. Moreover, advanced omics technologies are producing vast amount of genomic data with precision. Therefore, we are reporting annotated transcriptomic resources from *A. sapidissima* for the first time. For a migratory species, it’s a challenge to maintain the ionic-balance in body fluid at a steady-state as it requires a rhythmic alteration between solvent and solutes contents. Moreover, a well-developed signaling system is also required to switch from salt to fresh water and vice versa, and to feed live prey [15–18]. So, the current transcriptomic resource from blood and brain will aim to understand key biological features from molecular level for this precious species. Nevertheless, the resource was initially produced to compare with other shads, but the effort was halted due to biological material transfer incompatibilities during COVID-19 pandemic. Besides, WGS study of *A. sapidissimsa* is under consideration by the G10K consortium [19]. Thereafter, it would be useful to share the data with scientific community to make better use of it.

## Data description

A mature individual of 42 cm in SL was euthanized with MS222(1gL^− 1^) prior to extract brain and blood tissues, which were immediately placed in ALLProtect buffer and EDTA-stabilized anticoagulant tubes, respectively and later preserved in − 20 °C refrigerator [20]. Total RNA from each sample was extracted with TRIzol and 1 g was used to prepare cDNA libraries (~ 400 bp) for bridge amplification following the manufacturer’s instructions. Finally, the purified libraries were loaded into Illumina Novaseq with 2*150 bp paired-end configuration. Raw sequencing reads were trimmed where the base accuracy was strictly confined to 99.99% (Data file 5). To perform assembly, the processed reads were passed through Trinity-v2.11.0 [21, 22] assembler that constructed 195,742 and 158,817 transcripts from brain and blood samples, respectively (Data file 9). The primary number of transcripts was reduced to 160,481 and 129,040 after filtering and clustering non-redundant transcripts at 98% threshold. Quantitative analysis identified 41,572 bp and 17,242 bp from the brain and blood transcriptomes as the longest transcripts with N50 values of 2039 bp and 2096 bp (Data file 10). In both instances, the assembly length distribution remained uniform and comparable to one another (Data file 6). In addition, BUSCO searches against 3354 species from vertebrate lineages found 82.3% and 71.5% of complete universal single-copy genes from brain and blood transcriptomes (Data file 7).

Implication of TransDecoder-v5.5.0 [22] predicted around 80% of assembled transcripts had an ORF, of which 48,579 and 40,948 transcripts were capable of producing functional proteins (Data file 11). Using Blastx, Blastp as well as a series of tools based on HMM, we annotated coding and non-coding transcripts with an e value cut-off at 10^-5. GO analysis ascertained 39,015 and 33,475 proteins had at least one relevant term with molecular function, cellular component or biological process. In both instances, search against Pfam database revealed 70% of proteins with a functional domain. According to the loaded Sqlite database from Trinotate [23], 83% of predicted proteins were functionally annotated. Moreover, we made an assembly and subsequent annotation combining the reads from both tissues. The entire effort and representative datasets can be found in Table 1 (Data file 1, Data file 4 and Data file 14-20). To draw the homologous relationship, we retrieved Refseq proteins of seven other species, including clupeiform and non-clupeiform species from NCBI repository (Data file 12). For brain and blood, we found that 40,304 and 34,301 proteins had orthologue relationships with other species accounting for > 82% of total proteins (Data file 13). Finally, to evaluate the phylogenetic relationships, one-to-one orthologue proteins were retrieved. As the datasets from brain tissue extracted more groups of homologue proteins, we used 204 one-to-one orthologue proteins from brain to reconstruct a phylogenetic tree. We have found that *A. sapidissima* was clustered well with the clupeiform clade that was supported with maximum bootstrap value (Data file 8). The constructed phylogeny supports several other previous phylogenetic studies regarding their position [32–34]. However, this present resource will leverage the whole genome study of *A. sapidissima* as well as provide a solid foundation to compare their impressive physiological and behavioral competence with other allies.

**Table 1:** Overview of all data files/data sets

Label	Name of data file/data set	File types (file extensions)	Data repository and identifier (DOI or accession number)
Data file 1	Method and Code availability	Document file (.docx)	*Figshare* 10.6084/m9.figshare.17056328 [24]
Data file 2	RNAseq-Brain	SRA file (.sra)	NCBI *Sequence Read Archive* https://trace.ncbi.nlm.nih.gov/Traces/sra/?run=SRR16474177 [25]
Data file 3	RNAseq-Blood	SRA file (.sra)	NCBI *Sequence Read Archive* https://trace.ncbi.nlm.nih.gov/Traces/sra/?run=SRR16474180 [26]
Data file 4	FigS1 Complete work flow	Image file (.jpg)	*Figshare* 10.6084/m9.figshare.17054852 [27]
Data file 5	FigS2 Post trimming quality assessment	Image file (.jpg)	*Figshare* 10.6084/m9.figshare.17054852 [27]
Data file 6	FigS3 Transcript length distribution	Image file (.jpg)	*Figshare* 10.6084/m9.figshare.17054852 [27]
Data file 7	FigS4 BUSCO assessment	Image file (.jpg)	*Figshare* 10.6084/m9.figshare.17054852 [27]
Data file 8	FigS5 Phylogenetic relationship	Image file (.jpg)	*Figshare* 10.6084/m9.figshare.17054852 [27]
Data file 9	Table S1 Preliminary assembly statistics	Document file (.docx)	*Figshare* 10.6084/m9.figshare.17054948 [28]
Data file 10	Table S2 Final non-redundant assembly statistics	Document file (.docx)	*Figshare* 10.6084/m9.figshare.17054948 [28]
Data file 11	Table S3 Annotation summery	Document file (.docx)	*Figshare* 10.6084/m9.figshare.17054948 [28]
Data file 12	Table S4 Species description	Document file (.docx)	*Figshare* 10.6084/m9.figshare.17054948 [28]
Data file 13	Table S5 Homologue information	Document file (.docx)	*Figshare* 10.6084/m9.figshare.17054948 [28]
Data file 14	brain.Trinotate.filtered.xls	Spreadsheet (.xls)	*Figshare* 10.6084/m9.figshare.16834564.v2 [29]
Data file 15	brain.Trinity.RSEM.retained.clustered.fasta	Fasta file(.fasta)	*Figshare* 10.6084/m9.figshare.16834564.v2 [29]
Data file 16	brain.Trinity.RSEM.retained.clustered.fasta.transdecoder.pep	Fasta file(.pep)	*Figshare* 10.6084/m9.figshare.16834564.v2 [29]
Data file 17	blood.Trinotate.filtered.xls	Spreadsheet (.xls)	*Figshare* 10.6084/m9.figshare.16834546.v2 [30]
Data file 18	blood.Trinity.RSEM.retained.clustered.fasta	Fasta file(.fasta)	*Figshare* 10.6084/m9.figshare.16834546.v2 [30]
Data file 19	blood.Trinity.RSEM.retained.clustered.fasta.transdecoder.pep	Fasta file(.pep)	*Figshare* 10.6084/m9.figshare.16834546.v2 [30]
Data file 20	Annotation from combined reads	Document file (.docx)	*Figshare* 10.6084/m9.figshare.19308326 [31]

## Limitations

The sample was collected from freshwater captivity located at Songjiang District, Shanghai. Normally, when anadromous fish migrate to freshwater, they need to move against strong water currents and interact with particular abiotic factors. However, in captivity, possible absence of such physical properties might provide less chance to specific gene expression than during migration in the wild.

## Data Availability

Processed raw data has been deposited in NCBI with open access (https://trace.ncbi.nlm.nih.gov/Traces/sra/?run=SRR16474177 & https://trace.ncbi.nlm.nih.gov/Traces/sra/?run=SRR16474180*)*. Method with its codes and references and all the final product of analysis has been submitted to *figshare* for public usage [24–31]. File type and specific accessible links can be found in Table 1.

## Abbreviations

SL: Standard Length
BUSCO: Benchmarking Universal Single Copy Orthologs
ORF: Open Reading Frame
HMM: Hidden Markov Model
GO: Gene Ontology
NCBI: National Canter for Biotechnology Information
WGS: Whole Genome Study
G10K: The international Genome 10 K consortium
